# Salicylic Acid Mitigates the Effects of Water Deficit in Sour Passion Fruit in the Phenological Phases

**DOI:** 10.3390/plants14223507

**Published:** 2025-11-17

**Authors:** Allesson Ramos de Souza, Geovani Soares de Lima, André Alisson Rodrigues da Silva, Carlos Alberto Vieira de Azevedo, Lucyelly Dâmela Araújo Borborema, Kheila Gomes Nunes, Denis Soares Costa, Larissa Fernanda Souza Santos, Thiago Filipe de Lima Arruda, Luciano Marcelo Fallé Saboya, Lauriane Almeida dos Anjos Soares, Hans Raj Gheyi, Weslley Bruno Belo de Souza, Fellype Jonathar Lemos da Silva, Gustavo de Oliveira Porto

**Affiliations:** 1 Academic Unit of Agricultural Engineering, Federal University of Campina Grande, Campina Grande 58429-900, Brazil; carlos.vieira@professor.ufcg.edu.br (C.A.V.d.A.); lucyellyd@gmail.com (L.D.A.B.); kheilagomesnunes@gmail.com (K.G.N.); denis_soares11@hotmail.com (D.S.C.); thiago.filipe@estudante.ufcg.edu.br (T.F.d.L.A.); lsaboya@hotmail.com (L.M.F.S.); hans.gheyi@ufcg.edu.br (H.R.G.); 2 Academic Unit of Agrarian Sciences, Federal University of Campina Grande, Pombal 58840-000, Brazil; geovani.soares@professor.ufcg.edu.br (G.S.d.L.); englarissafss@gmail.com (L.F.S.S.); laurispo.agronomia@gmail.com (L.A.d.A.S.); weslleybruno56@gmail.com (W.B.B.d.S.); 3 Academic Unit of Agronomy, Universidade Federal do Oeste do Pará, Juruti 68170-000, Brazil; andrealisson_cgpb@hotmail.com (A.A.R.d.S.); fellype.jonathar@estudante.ufcg.edu.br (F.J.L.d.S.); 4 Academic Ecology and Conservation, Universidade Estadual da Paraíba, Campina Grande 58429-500, Brazil; gustavo.oliveira.porto@aluno.uepb.edu.br

**Keywords:** *Passiflora edulis* Sims, semi-arid, abiotic stress, phytohormone

## Abstract

Practices that mitigate the deleterious effects of water deficit are of great importance for agricultural production in the semi-arid region of Northeastern Brazil. The objective of this study was to evaluate the effect of foliar application of salicylic acid on mitigating water deficit in the morphophysiology and yield components of sour passion fruit during different phenological stages. Treatments were arranged in a randomized block design in a 6 × 2 factorial scheme, consisting of six irrigation strategies under water deficit, based on reference evapotranspiration (ETr) [irrigation with 100% ETr throughout the entire cultivation cycle—IS1; irrigation with 50% ETr during the vegetative stage—IS2; flowering—IS3; fruiting—IS4; vegetative/flowering—IS5; and vegetative/fruiting—IS6], combined with or without salicylic acid (SA) application (0 and 1 mM). The application of 1.0 mM salicylic acid alleviated the effects of water deficit during the flowering stage, improving gas exchange, photochemical efficiency, relative water content, growth, and yield of sour passion fruit. In addition, it contributed to reducing electrolyte leakage in the leaf blade. Therefore, foliar application of salicylic acid represents a promising strategy to maintain the integrity of the photosynthetic apparatus and the productive performance of sour passion fruit under water deficit conditions.

## 1. Introduction

The passion fruit (*Passiflora edulis* Sims) is a tropical crop native to Brazil [1]. Its fruit is characterized by being rich in vitamins, polyphenols, flavonoids, lipids, and antioxidant compounds, which may be beneficial in the treatment of chronic degenerative diseases [2]. Brazil is recognized as the world’s largest producer and exporter of passion fruit [3]. The Northeastern region of the country, in turn, is the largest producer, accounting for 69.77% of the 607,859 tons produced nationwide in 2022. Despite this representativeness, the regional average yield (14.76 kg per hectare) remains lower than that of other regions of the country, reflecting the limitations imposed by the semiarid climate, characterized by irregular rainfall and high evapotranspiration, which affect water availability and crop production [4,5].

When exposed to water deficit conditions, plants respond by partially closing their stomata, which reduces water loss to the atmosphere and decreases CO_2_ diffusion to RuBisCO, resulting in a lower photosynthetic rate [6]. The amount of water present in the soil and the availability of nutrients are strongly correlated with the level of water stress, reinforcing the essential role of water in determining crop productivity [7]. Although it is well established that water plays a vital role in plant development, the details of the underlying response mechanisms have not yet been fully elucidated [8]. According to Ferreira et al. [9], plant sensitivity to stress may vary depending on phenological stages, genotype, edaphoclimatic conditions, and irrigation and fertilization practices.

Studies conducted by Fatima et al. [10] elucidated that the sensitivity of sour passion fruit to water deficit may vary according to the developmental stage. It is also noteworthy that stress during the flowering stage resulted in greater physiological acclimation, although it caused more pronounced yield losses. In this context, elucidating plant responses to water deficit, as well as developing strategies aimed at mitigating its harmful effects while simultaneously promoting efficient water use in sour passion fruit cultivation under semi-arid conditions, is of great importance. It is also worth considering the use of elicitor substances, such as salicylic acid (SA), as a promising strategy. Salicylic acid (SA) is a phytohormone involved in the regulation of physiological and biochemical processes related to tolerance to abiotic stresses [11], including stomatal regulation, enhanced water uptake efficiency, photochemical efficiency, and plant growth [12], which are important parameters affected under water scarcity conditions.

The beneficial effect of salicylic acid in different plant species under abiotic stresses has been attributed to its role in the activity of antioxidant enzymes and in protecting against damage to cell membranes [13]. Depending on its concentration, salicylic acid can act as a signaling molecule, promoting greater induction of tolerance to abiotic stresses in passion fruit crops [14,15]. Previous studies have reported that salicylic acid mitigated the deleterious effects of water deficit [14] on the morphophysiology of passion fruit seedlings, in addition to significantly increasing relative water content, photosynthetic pigment levels, and fruit yield in plants irrigated with saline water [16]. However, studies evaluating the application of salicylic acid in mitigating the negative effects of water deficiency during different phenological stages of *Passiflora edulis* cv. Redondo Amarelo remain scarce in the literature.

In general, considering that water deficit is one of the most limiting factors for the production of sour passion fruit, especially in the semiarid regions of Northeastern Brazil, this study was based on the hypothesis that foliar application of salicylic acid mitigates the negative effects of water deficit on the morphophysiology and yield components of sour passion fruit by enhancing the activity of antioxidant enzymes and regulating photosynthetic activity.

Accordingly, the objective of the present study was to evaluate the effect of foliar application of salicylic acid on mitigating water deficit stress in the morphophysiology and yield components of sour passion fruit during different phenological stages under semiarid conditions.

## 2. Results

An interaction effect between the factors (Irrigation Strategy × Salicylic Acid) was observed for the relative water content and electrolyte leakage of sour passion fruit plants (Table 1).

**Figure 1:**
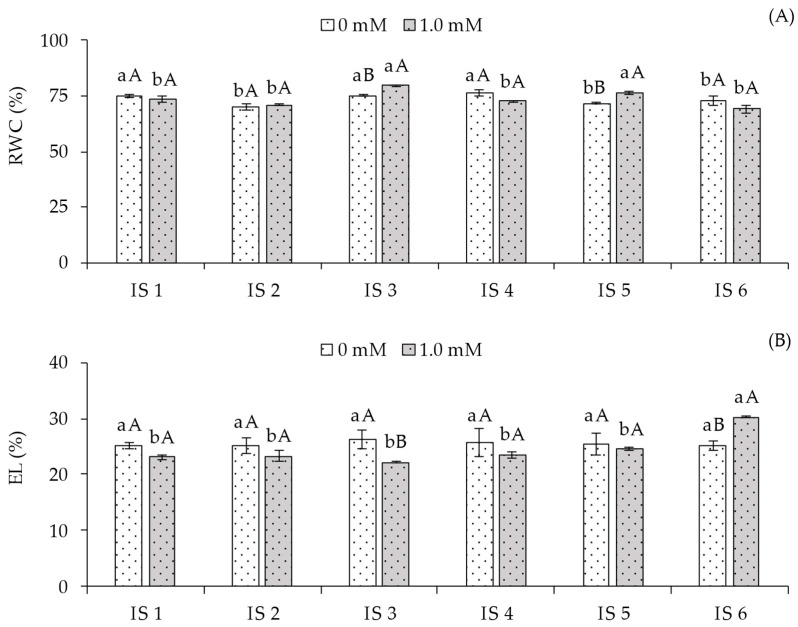
Relative water content—RWC% (**A**) and electrolyte leakage—EL% (**B**) of sour passion fruit cv. Redondo Amarelo, as affected by the interaction between irrigation strategies under water deficit and salicylic acid application, at 160 days after transplanting. The same lowercase letters indicate no significant difference among irrigation strategies under water deficit within each salicylic acid concentration (Scott-Knott test, *p* ≤ 0.05), while the same uppercase letters indicate no significant difference between salicylic acid concentrations within each irrigation strategy under water deficit (Tukey test, *p* ≤ 0.05). Vertical bars represent the standard error of the mean (n = 4). IS1—no water deficit throughout the crop cycle; IS2, IS3, IS4, IS5, and IS6—water deficit applied during the vegetative, flowering, fruiting, vegetative/flowering, and vegetative/fruiting stages, respectively.

The relative water content (RWC) (Figure 1A) of plants grown without foliar application of salicylic acid showed the highest values (75.10, 75.20, and 76.20%) under irrigation strategies IS1, IS3, and IS4, differing significantly from those irrigated with 50% ETr during the vegetative/flowering (IS5) and vegetative/fruiting (IS6) stages. When analyzing the effect of SA concentrations within each irrigation strategy, plants subjected to IS3 and IS5 with foliar application of 1.0 mM SA exhibited statistically higher RWC compared to those grown without SA in the respective treatments. For the other irrigation strategies, no significant differences were observed between SA concentrations.

Regarding electrolyte leakage (EL) in the leaf blade (Figure 1B) of sour passion fruit plants that did not receive salicylic acid application (0.0 mM), no significant differences were observed among the irrigation strategies. However, the 1.0 mM SA concentration in plants irrigated with 50% ETr during the vegetative/fruiting stage (IS6) differed statistically from the other strategies, where SA application resulted in higher EL% in plants under water deficit during the vegetative/fruiting stages.

A significant effect was observed for the interaction between the different irrigation strategies under water deficit (IS) and salicylic acid (SA) concentrations on stomatal conductance (*gs*), transpiration rate (*E*), CO_2_ assimilation rate (*A*), instantaneous carboxylation efficiency (*CEi*), and instantaneous water use efficiency (*WUEi*) (Table 2). However, internal carbon concentration (*Ci*) was influenced only by the application of salicylic acid.

**Figure 2:**
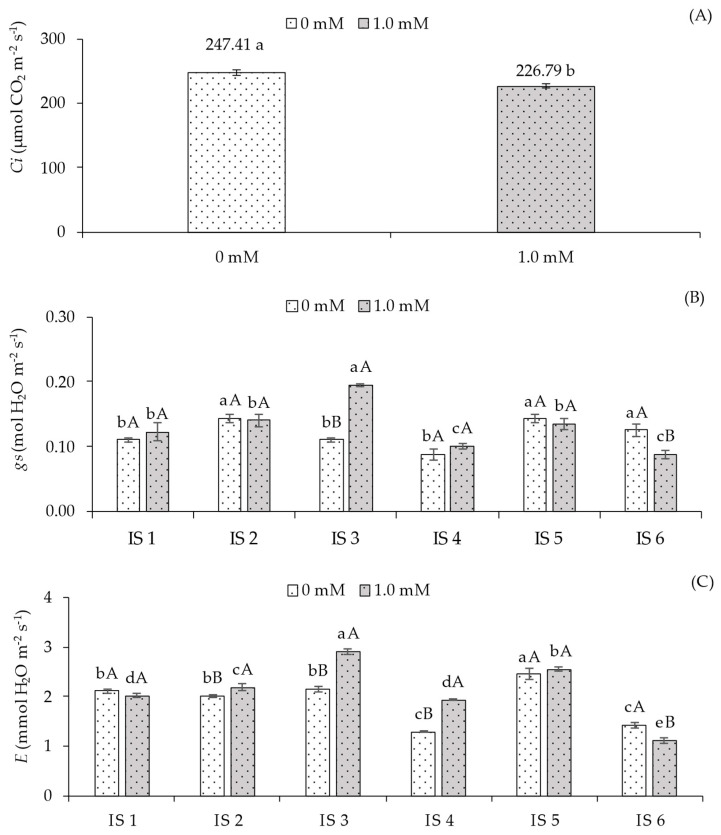

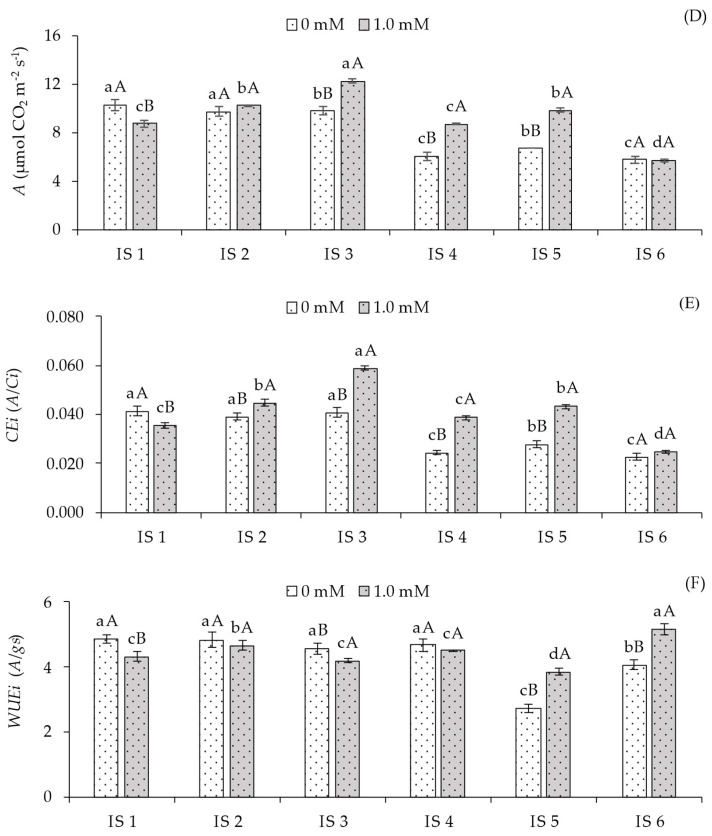
Intercellular CO_2_ concentration—*Ci* (**A**) as a function of salicylic acid concentrations, stomatal conductance—*gs* (**B**), transpiration rate—*E* (**C**), CO_2_ assimilation rate—A (**D**), instantaneous carboxylation efficiency—*CEi* (**E**), and instantaneous water use efficiency—*WUEi* (**F**) of sour passion fruit cv. Redondo Amarelo, as affected by the interaction between irrigation strategies under water deficit and salicylic acid application, at 160 days after transplanting. The same lowercase letters indicate no significant difference among irrigation strategies under water deficit within each salicylic acid concentration (Scott–Knott test, *p* ≤ 0.05), while the same uppercase letters indicate no significant difference between salicylic acid concentrations within each irrigation strategy under water deficit (Tukey test, *p* ≤ 0.05). Vertical bars represent the standard error of the mean (n = 4). IS1—no water deficit throughout the crop cycle; IS2, IS3, IS4, IS5, and IS6—water deficit applied during the vegetative, flowering, fruiting, vegetative/flowering, and vegetative/fruiting stages, respectively.

Internal carbon concentration (*Ci*) (Figure 2A) was influenced by salicylic acid application, with the lowest value of 226.79 µmol CO_2_ m^−2^ s^−1^ observed in sour passion fruit plants treated with 1.0 mM foliar SA, representing a reduction of 9.09% (20.62 µmol CO_2_ m^−2^ s^−1^) compared to plants that did not receive SA application.

For stomatal conductance (*gs*) (Figure 2B), plants grown without SA application and subjected to water deficit during the vegetative (IS2), vegetative/flowering (IS5), and vegetative/fruiting (IS6) stages showed the highest values (0.14, 0.13, and 0.15 mol H_2_O m^−2^ s^−1^), differing statistically from those irrigated with 100% ETr throughout the cycle (IS1) and with water deficit during the flowering (IS3) and fruiting (IS4) stages. In plants receiving salicylic acid application, significant differences were observed in treatments IS3 and IS6. The 1.0 mM SA concentration resulted in a 43.69% increase (0.08 mol H_2_O m^−2^ s^−1^) in *gs* for plants under water deficit during flowering compared to plants without SA application (0.0 mM). Conversely, SA application in plants subjected to water deficit during the vegetative/fruiting stage led to a 42.86% reduction compared to those without SA.

Regarding transpiration (E) (Figure 2C), plants grown without SA application under water deficit during the vegetative/flowering stage showed the highest E (2.45 mmol H_2_O m^−2^ s^−1^), differing from the other irrigation strategies. SA application in plants under full irrigation (100% ETr) throughout the cycle (IS1) did not result in significant differences in E. However, the application of 1.0 mM SA promoted significant increases in this variable for plants irrigated with deficit during the vegetative (IS2), flowering (IS3), and fruiting (IS4) stages, with the highest value (2.91 mmol H_2_O m^−2^ s^−1^) observed in IS3 compared to plants that did not receive SA application.

For the CO_2_ assimilation rate (A) (Figure 2D), in the absence of SA application, plants grown under full irrigation (100% ETr) throughout the cycle (IS1) and under water deficit during the vegetative (IS2) and flowering (IS3) stages differed significantly from those subjected to treatments IS4, IS5, and IS6. However, foliar application of 1.0 mM salicylic acid promoted positive responses in plants subjected only to strategies IS3, IS4, and IS5 compared to those grown without SA application.

Water deficit (50% ETr) in plants grown without SA application (0.0 mM) significantly reduced instantaneous carboxylation efficiency (*CEi*) during the fruiting (IS4), vegetative/flowering (IS5), and vegetative/fruiting (IS6) stages compared to plants under full irrigation (IS1). When analyzing the effect of SA application across different irrigation strategies, no significant differences were observed only for plants irrigated under deficit during the vegetative/fruiting stage (IS6). Conversely, the 1.0 mM SA concentration increased *CEi* in plants subjected to water deficit in treatments IS2, IS3, IS4, and IS5 compared to plants grown under the same water conditions without SA application. On the other hand, under full irrigation (100% ETr), plants without SA application exhibited statistically higher *CEi* compared to those treated with 1.0 mM SA.

Instantaneous water use efficiency (*WUEi*) (Figure 2F) of sour passion fruit plants grown without foliar SA application (0.0 mM) showed the highest values of 4.56, 4.82, 4.56, and 4.67 [(µmol CO_2_ m^−2^ s^−1^) (mol H_2_O m^−2^ s^−1^)^−1^] under irrigation strategies IS1, IS2, IS3, and IS4, differing significantly from those grown under water deficit during the vegetative/flowering (IS5) and vegetative/fruiting (IS6) stages. In plants without SA application, *WUEi* was statistically higher compared to those treated with 1.0 mM SA in irrigation strategies IS1 and IS3. For strategies IS2 and IS4, no significant differences were observed between SA concentrations. Conversely, the application of 1.0 mM SA significantly increased *WUEi* compared to plants without SA in irrigation strategies IS5 and IS6.

A significant interaction effect between deficit irrigation strategies (IS) and salicylic acid (SA) concentrations (*p* ≤ 0.05) was observed for chlorophyll *a* (Chl _a_), total chlorophyll (Chl total), and carotenoid (Car) contents (Table 3).

**Figure 3:**
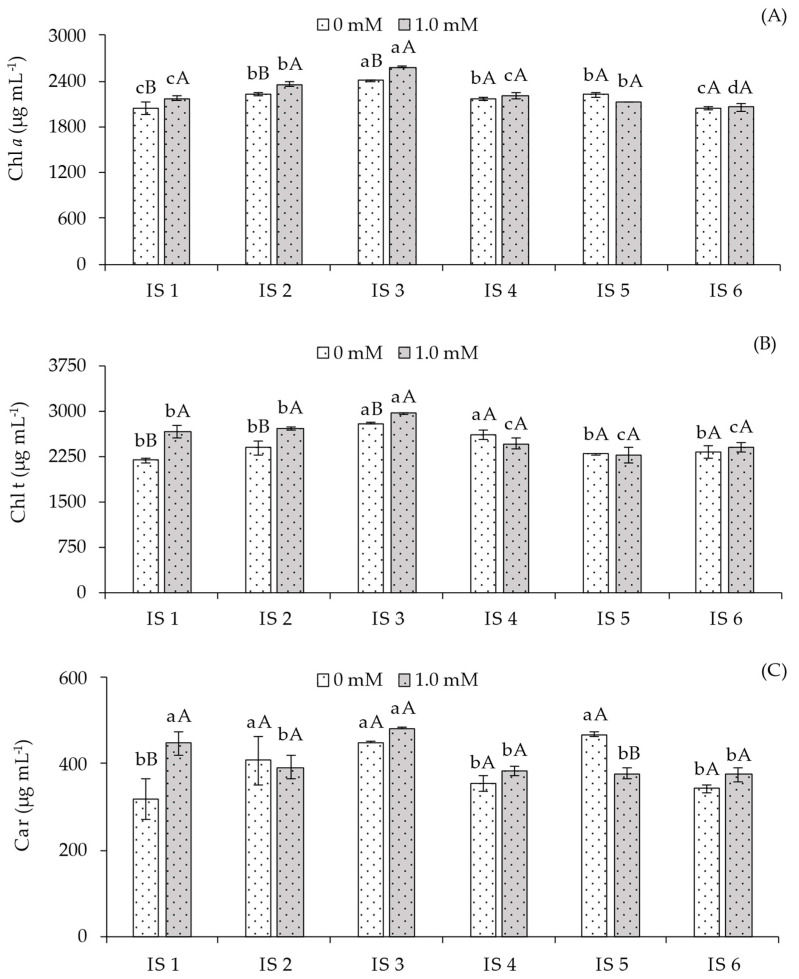
Chlorophyll *a*—Chl *a* (**A**), total chlorophyll—Chl t (**B**), and carotenoids—Car (**C**) of sour passion fruit cv. Redondo Amarelo, as affected by the interaction between irrigation strategies under water deficit and salicylic acid application, at 160 days after transplanting. The same lowercase letters indicate no significant difference among irrigation strategies under water deficit within each salicylic acid concentration (Scott–Knott test, *p* ≤ 0.05), while the same uppercase letters indicate no significant difference between salicylic acid concentrations within each irrigation strategy under water deficit (Tukey test, *p* ≤ 0.05). Vertical bars represent the standard error of the mean (n = 4). IS1—no water deficit throughout the crop cycle; IS2, IS3, IS4, IS5, and IS6—water deficit applied during the vegetative, flowering, fruiting, vegetative/flowering, and vegetative/fruiting stages, respectively.

Regarding chlorophyll *a* (Chl *a*) content (Figure 3A), in plants not subjected to salicylic acid application, the highest value (2405.00 μg mL^−1^) was observed under water deficit during the flowering stage (IS3), differing significantly from the other irrigation strategies. In plants under treatments IS2, IS4, and IS5, Chl *a* content was statistically higher compared to those grown under IS1 and IS6. Conversely, plants receiving foliar application of 1.0 mM salicylic acid during the flowering stage (IS3) exhibited the highest Chl *a* content, which was statistically higher than plants under IS1, IS2, IS4, IS5, and IS6. At this concentration, no significant differences in Chl *a* content were observed between plants under IS2 and IS5. When analyzing the effect of SA concentrations within each irrigation strategy, significant differences were observed in treatments IS1, IS2, and IS3. For the remaining strategies, no significant differences were observed between SA concentrations.

For total chlorophyll content (Chl t) (Figure 3B), plants subjected to water deficit during the flowering (IS3) and fruiting (IS4) stages exhibited the highest values among the strategies without SA application, compared to full irrigation (100% ETr). The other strategies did not differ statistically. In plants receiving 1.0 mM foliar SA, water deficit during the flowering stage (IS3) resulted in the highest Chl t content, differing significantly from plants under IS1, IS2, IS4, IS5, and IS6. When analyzing the effect of SA concentrations within each irrigation strategy, the application of 1.0 mM SA resulted in statistically higher Chl total content compared to plants without SA in strategies IS1, IS2, and IS3. For the remaining irrigation strategies, no significant differences were observed between SA concentrations.

Carotenoid content (Car) (Figure 3C) was increased under water deficit during the vegetative (IS1), flowering (IS3), and vegetative/flowering (IS5) stages, but reduced under 50% ETr irrigation during fruiting (IS4) and vegetative/fruiting (IS6). Significant differences between salicylic acid concentrations were observed only in plants under full irrigation (100% ETr) throughout the cycle and water deficit during the vegetative/flowering stage (IS5). However, SA application benefited plants irrigated with 100% ETr throughout the cycle compared to the control treatment (0 mM SA). For the remaining strategies, no significant differences were observed between plants with and without SA application.

A significant interaction effect between the factors (IS × SA) was observed only for maximum fluorescence (Fm) (Table 4). Salicylic acid significantly influenced only the initial fluorescence (F_o_), while the irrigation strategies significantly affected (*p* ≤ 0.05) variable fluorescence (Fv) and the quantum efficiency of photosystem II (Fv/Fm).

**Figure 4:**
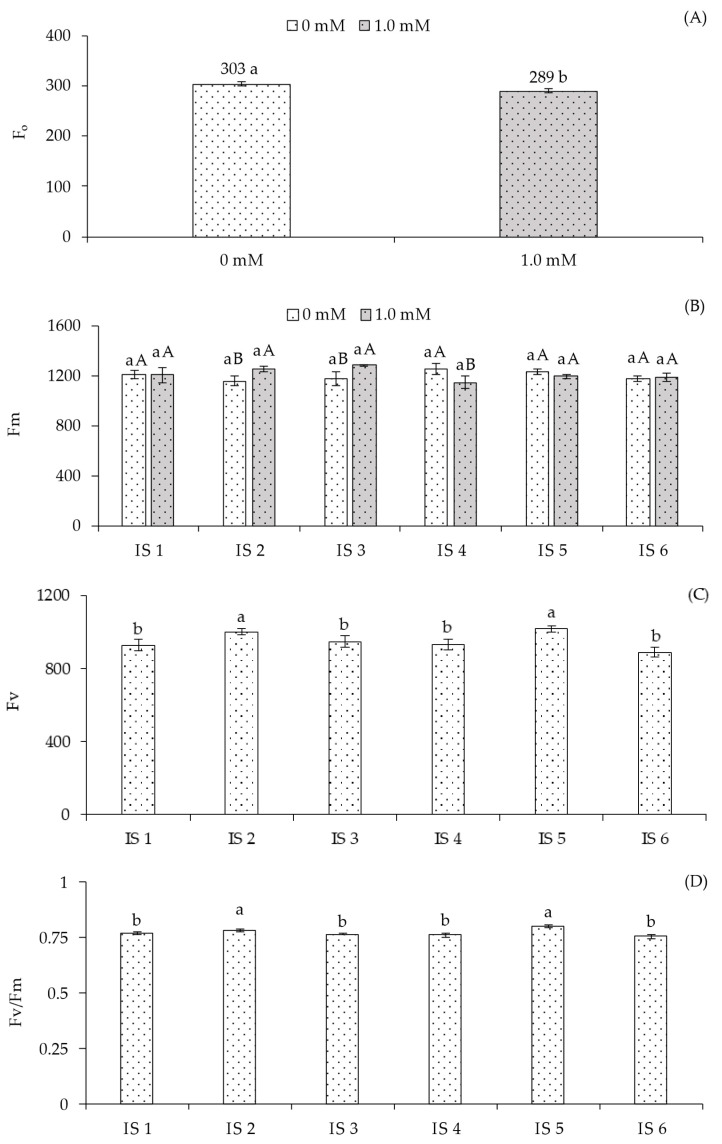
Initial fluorescence—F_0_ (**A**) as a function of salicylic acid concentrations, maximum fluorescence—Fm (**B**) as affected by the interaction between irrigation strategies under water deficit and salicylic acid application, variable fluorescence—Fv (**C**), and quantum efficiency of photosystem II—Fv/Fm (**D**) as a function of irrigation strategies under water deficit of sour passion fruit cv. Redondo Amarelo, at 160 days after transplanting. The same lowercase letters indicate no significant difference among irrigation strategies under water deficit within each salicylic acid concentration (Scott-Knott test, *p* ≤ 0.05), while the same uppercase letters indicate no significant difference between salicylic acid concentrations within each irrigation strategy under water deficit (Tukey test, *p* ≤ 0.05). Vertical bars represent the standard error of the mean (n = 4). IS1—no water deficit throughout the crop cycle; IS2, IS3, IS4, IS5, and IS6—water deficit applied during the vegetative, flowering, fruiting, vegetative/flowering, and vegetative/fruiting stages, respectively.

The application of salicylic acid (1.0 mM) resulted in the lowest fluorescence value (289.00) (Figure 4A), differing significantly from plants grown without foliar application of this phytohormone (0 mM). When analyzing maximum fluorescence (Fm) (Figure 4B) of sour passion fruit under different irrigation strategies without salicylic acid application, no significant differences were observed. However, when evaluating the effect of SA concentrations within each irrigation strategy, foliar application of 1.0 mM SA resulted in statistically higher Fm compared to plants without SA in strategies IS2 and IS3. For the remaining irrigation strategies, no significant differences were observed between the different SA applications.

Variable fluorescence (Fv) (Figure 4C) was significantly influenced by irrigation strategies (IS). Plants subjected to water deficit during the vegetative (IS2) and vegetative/flowering (IS5) stages showed the highest mean values of 1002.12 and 1019.63, respectively, which were statistically higher than those grown under IS1, IS3, IS4, and IS6. No significant differences were observed among plants under IS1, IS3, IS4, and IS6. Regarding the quantum efficiency of photosystem II (Fv/Fm) (Figure 4D), plants subjected to water deficit during the vegetative (IS2) and vegetative/flowering (IS5) stages showed higher PSII efficiency, with increases of 1.45% and 4.29%, respectively, compared to plants receiving 100% water replacement (IS1). However, no significant differences in Fv/Fm were observed among plants under IS1, IS3, IS4, and IS6.

According to Table 5, a significant interaction effect between the factors (IS × SA) was observed only for the diameter of the main stem (SD) and total yield (TY). The average diameter of secondary branches (SBD) was influenced (*p* ≤ 0.05) only by salicylic acid concentrations.

**Figure 5:**
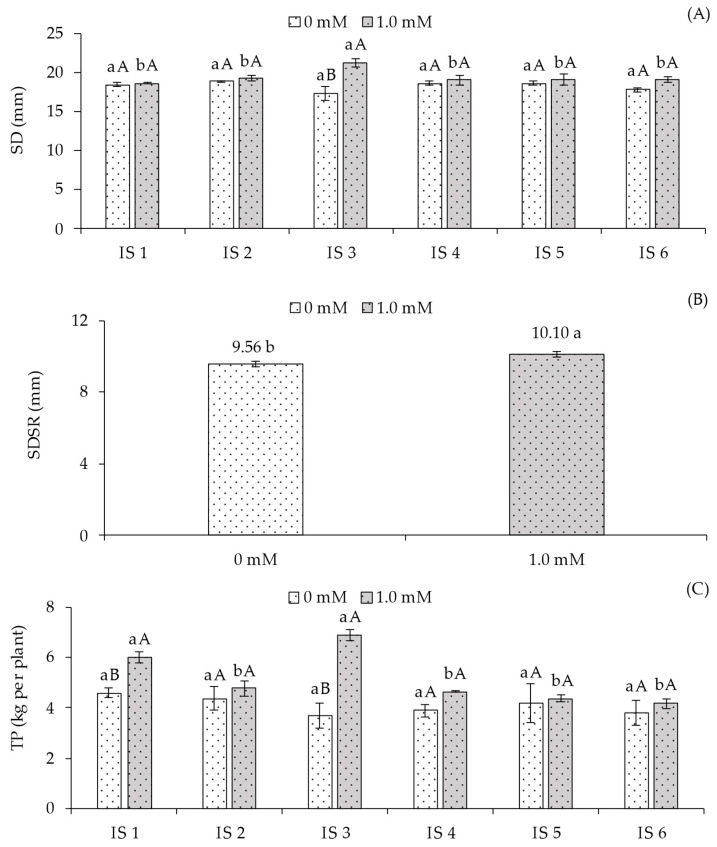
Stem diameter of the main branch—SD (**A**), and production per plant—PT (**C**) of the sour passion fruit cv. Redondo Amarelo, as a function of the interaction between irrigation strategies with water deficit and the application of salicylic acid, and average diameter of secondary branches—SDSR (**B**) as a function of salicylic acid concentrations, 160 days after transplanting. The same lowercase letters indicate no significant difference among irrigation strategies under water deficit within each salicylic acid concentration (Scott-Knott test, *p* ≤ 0.05), while the same uppercase letters indicate no significant difference between salicylic acid concentrations within each irrigation strategy under water deficit (Tukey test, *p* ≤ 0.05). Vertical bars represent the standard error of the mean (n = 4). IS1—no water deficit throughout the crop cycle; IS2, IS3, IS4, IS5, and IS6—water deficit applied during the vegetative, flowering, fruiting, vegetative/flowering, and vegetative/fruiting stages, respectively.

The stem diameter (SD) (Figure 5A) of sour passion fruit plants was not significantly affected by irrigation strategies when grown without foliar salicylic acid application. In plants receiving foliar SA, water deficit during the flowering stage (IS3) resulted in a statistically higher SD compared to IS1, IS2, IS4, IS5, and IS6. When analyzing SA concentrations within each irrigation strategy, plants treated with 1.0 mM SA under water deficit during flowering exhibited higher SD compared to those without SA application (0 mM). In the remaining irrigation strategies, SA application did not produce significant effects in any other treatment.

The average diameter of secondary branches (SBD) (Figure 5B) was significantly influenced by salicylic acid application. The 1.0 mM concentration resulted in a statistically higher value (10.10 cm), representing an increase of 5.65% compared to plants not receiving SA application (0.0 mM).

A pattern similar to that observed for electrolyte leakage (Figure 1B), maximum fluorescence (Figure 4B), and stem diameter (Figure 5A) was found for the yield (Figure 5C) of sour passion fruit, with no significant differences among irrigation strategies in the absence of salicylic acid application. However, considering the interaction between SA concentrations within each strategy, plants receiving 1.0 mM exhibited the highest yield values: 6.00 kg in IS1 (100% ETr throughout the cycle) and 6.88 kg in IS3 (water deficit during the flowering stage). Compared to plants under the same water conditions but without SA application, the corresponding increases were 30.78% and 87.21%, respectively.

**Figure 6:**
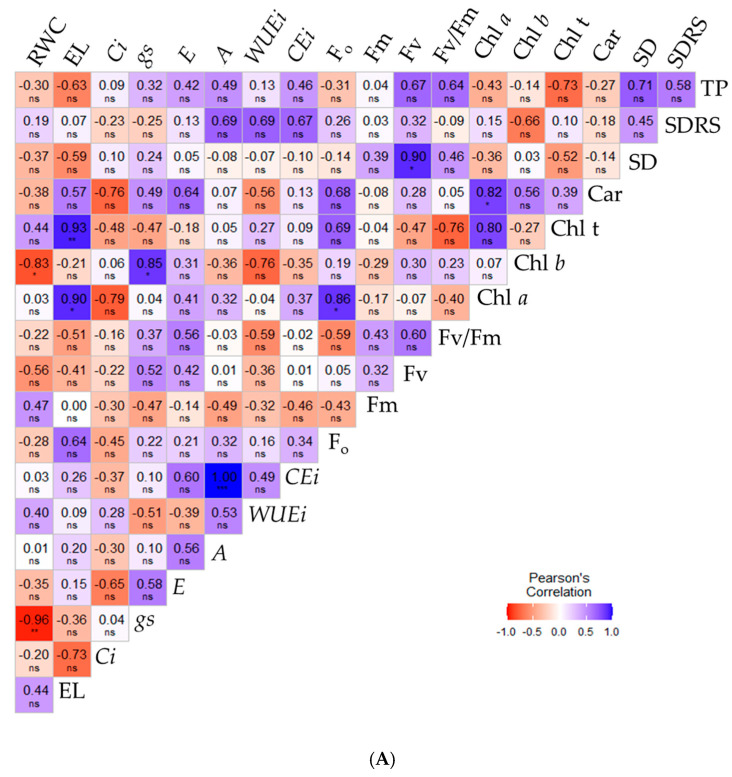

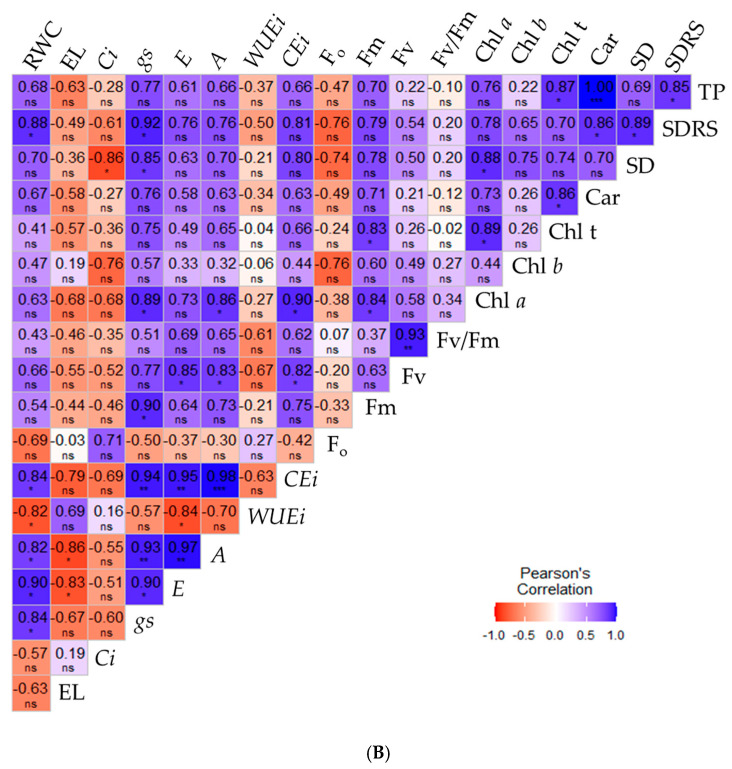
Pearson correlation matrix for the variables analyzed within the irrigation strategies and salicylic acid concentrations of 0.0 mM (**A**) and 1.0 mM (**B**), for the variables RWC (%), EL (%), *Ci* (µmol CO_2_ m^−2^ s^−1^), *gs* (mol H_2_O m^−2^ s^−1^), *E* (mol H_2_O m^−2^ s^−1^), *A* (µmol CO_2_ m^−2^ s^−1^), *CEi* (*A*/*Ci*), *WUEi* (*A*/*E*), Chl *a* (μg mL^−1^), Chl *b* (μg mL^−1^), Chl t (μg mL^−1^), Car (μg mL^−1^), Fo, Fm, Fv, Fv/Fm, SD (mm), SDRS (mm), TP (kg per plant). Relative water content—RWC (%), electrolyte leakage—EL (%), intercellular CO_2_ concentration—*Ci* (µmol CO_2_ m^−2^ s^−1^), stomatal conductance—*gs* (mol H_2_O m^−2^ s^−1^), transpiration rate—*E* (mol H_2_O m^−2^ s^−1^), CO_2_ assimilation rate—*A* (µmol CO_2_ m^−2^ s^−1^), instantaneous carboxylation efficiency—*CEi* (*A*/*Ci*), instantaneous water use efficiency—*WUEi* (*A*/*E*), chlorophyll *a*—Chl *a* (μg mL^−1^), chlorophyll *b*—Chl *b* (μg mL^−1^), total chlorophyll—Chl t (μg mL^−1^), carotenoids—Car (μg mL^−1^), initial fluorescence—Fo, maximum fluorescence—Fm, variable fluorescence—Fv, quantum efficiency of photosystem II—Fv/Fm, diameter of the stem of the main branch—SD (mm), mean diameter of the secondary branches—SDRS (mm), production per plant—TP (kg per plant). *, **, ***, ^ns^—Significant at *p* ≤ 0.05, *p* ≤ 0.01, and not significant, respectively according to the f-test.

Changes in physiological, growth, and yield variables of sour passion fruit plants cultivated without application (0.0 mM) and with 1.0 mM SA can be observed in the Pearson correlation matrices (Figure 6A,B). Fewer significant correlations (*p* ≤ 0.05) were observed for plants under irrigation strategies without salicylic acid application (Figure 6A). Relative water content was strongly negatively correlated with chlorophyll *b* (−0.83) and stomatal conductance (−0.96), but showed no correlation with the other variables. Conversely, *gs* and *A* were the only gas exchange variables exhibiting strong positive correlations of 0.85 and 1.00 with chlorophyll *b* and instantaneous carboxylation efficiency, respectively. For the remaining variables, a few significant correlations were observed.

However, when sour passion fruit plants were subjected to foliar applications of 1.0 mM salicylic acid (Figure 6B), a higher correlation index among the variables was observed. Relative water content (RWC) showed positive correlations with most variables, except for instantaneous water use efficiency, intercellular carbon concentration, and electrolyte leakage. Nevertheless, RWC (%) exhibited significant, strong positive correlations (*p* ≤ 0.05) only with *gs* (0.84), *E* (0.90), *A* (0.82), and SD (0.88).

Electrolyte leakage in the leaf blade showed strong negative correlations with transpiration (E) and CO_2_ assimilation rate (A), with values of −0.83 and −0.86, respectively. All gas exchange variables were positively correlated with each other, except for intercellular CO_2_ concentration (*Ci*), which showed no significant correlation with the other variables, except for stem diameter, with which it had a strong negative correlation (−0.86). Regarding photosynthetic efficiency, variable fluorescence showed strong positive correlations (r > 0.80) with *E*, *A*, and *CEi*. Total fruit yield was positively correlated only with carotenoids (0.85) and SD (1.00) in sour passion fruit plants.

**Figure 7:**
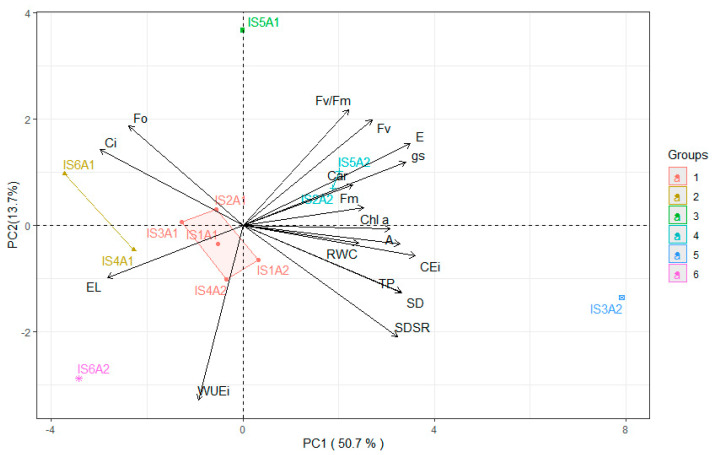
Two-dimensional projection of the principal component scores for deficit irrigation strategies (IS) and salicylic acid (SA) concentrations, showing the distribution of the analyzed variables across the two principal components (PC1 and PC2). Relative water content—RWC (%), electrolyte leakage—EL (%), intercellular CO_2_ concentration—*Ci* (µmol CO_2_ m^−2^ s^−1^), stomatal conductance—*gs* (mol H_2_O m^−2^ s^−1^), transpiration rate—*E* (mol H_2_O m^−2^ s^−1^), CO_2_ assimilation rate—*A* (µmol CO_2_ m^−2^ s^−1^), instantaneous carboxylation efficiency—*CEi* (*A*/*Ci*), instantaneous water use efficiency—*WUEi* (*A*/*E*), chlorophyll *a*—Chl *a* (μg mL^−1^), carotenoids—Car (μg mL^−1^), initial fluorescence—Fo, maximum fluorescence—Fm, variable fluorescence—Fv, quantum efficiency of photosystem II—Fv/Fm, diameter of the stem of the main branch—SD (mm), mean diameter of the secondary branches—SDRS (mm), production per plant—TP (kg per plant). IS1A1 (no water deficit throughout the crop cycle and 0 mM salicylic acid); IS2A1(water deficit applied during the vegetative stage and 0 mM salicylic acid); IS3A1(water deficit applied during the flowering stage and 0 mM salicylic acid); IS4A1(water deficit applied during the fruiting stage and 0 mM salicylic acid); IS5A1(water deficit applied during the vegetative/flowering stage and 0 mM salicylic acid); IS6A1(water deficit applied during the vegetative/fruiting stage and 1.0 mM salicylic acid); IS1A2 (no water deficit throughout the crop cycle and 1.0 mM salicylic acid); IS2A2(water deficit applied during the vegetative stage and 1.0 mM salicylic acid); IS3A2(water deficit applied during the flowering stage and 1.0 mM salicylic acid); IS4A2(water deficit applied during the fruiting stage and 1.0 mM salicylic acid); IS5A2(water deficit applied during the vegetative/flowering stage and 1.0 mM salicylic acid) and IS6A2(water deficit applied during the vegetative/fruiting stage and 1.0 mM salicylic acid).

The interaction between deficit irrigation strategies (IS) and salicylic acid (SA) concentrations can be observed in the principal component analysis (Figure 7). The PC accounted for 64.40% of the original variation, corresponding to the two components (PC1 and PC2), which individually explained 50.70% and 13.70% of the variance, respectively. The variables RWC, EL, *Ci*, *gs*, *E*, *A*, *CEi*, F_o_, Fm, Fv, Chl *a*, Car, SD, SDSR, and TP were retained in the first component. Negative correlations were observed only for electrolyte leakage (−0.67), internal carbon concentration (−0.75), and initial chlorophyll a fluorescence (−0.60), which were favored by cluster 2. For the remaining variables, correlations were higher than 0.60, showing greater clustering in groups 4 and 5.

The second component was composed solely of the variables *WUEi* (0.81) and Fv/Fm (−0.66), which were favored only by groups 1 and 3.

## 3. Discussion

In semiarid regions, the irregularity and poor distribution of rainfall, combined with high evapotranspiration rates and temperatures, result in water scarcity during most of the year. The reduction in water availability is a limiting factor for agricultural production and stands out as one of the main abiotic stresses affecting plant physiology and development. According to the United Nations Convention to Combat Desertification (UNCCD) report, by 2050, drought could affect more than three-quarters of the global population, potentially causing a series of disasters, including reduced agricultural productivity [17]. In this context, the transition from full irrigation to deficit irrigation has become an important strategy in countries with arid and/or semiarid climates to conserve water resources [18]. Therefore, practices that can mitigate the negative effects of this stress and/or promote greater acclimation to such conditions are extremely important. The foliar application of compatible solutes, such as salicylic acid (SA), represents a strategy that can play a crucial role in mitigating and/or inducing tolerance to the negative impacts of water deficit [19].

The deleterious effects of water deficit are associated with a reduction in cellular water content [20], inhibition of plant growth [21], and the induction of oxidative stress resulting from the excessive production of reactive oxygen species (ROS). In sour passion fruit (*Passiflora edulis f.*), water deficit can lead to a reduction in leaf area, fresh biomass, photosynthetic pigment content, and stomatal conductance [10,14,22].

In the present study, deficit irrigation (50% ETr) during the vegetative (IS2), vegetative/flowering (IS5), and vegetative/fruiting (IS6) phases negatively affected the relative water content (Figure 1A), which may have caused partial stomatal closure (Figure 2B) and a decrease in the transpiration rate (Figure 2C), reducing the release of water vapor to the atmosphere. With a smaller stomatal aperture, there was a tendency for reduced CO_2_ accumulation in the substomatal chamber, which may have resulted in a lower CO_2_ assimilation rate (Figure 2D). It is important to note that these variables showed a strong positive correlation (Figure 7), supporting the hypothesis that they are directly proportional, with water deficit in IS2, IS5, and IS6 exerting a negative effect on them.

Under ideal CO_2_ conditions, the carboxylation process of ribulose-1,5-bisphosphate (RuBisCO) prevails over the oxygenation process. However, under deficit irrigation, a reduction in photosynthetic activity is observed in sour passion fruit (*Passiflora edulis f. flavicarpa*) plants. The decrease in *A* may be associated with reduced carboxylase activity of RuBisCO, with a predominance of oxygenase activity, an effect potentially favored by lower *gs* values in plants subjected to water deficit [23].

Nevertheless, the application of salicylic acid (SA) showed beneficial effects on relative water content (RWC) (Figure 1A) and a decrease in electrolyte leakage (EL) (Figure 1B), especially when SA was applied in combination with deficit irrigation during the flowering stage (IS3). The increase in RWC due to SA application is possibly related to its role in enhancing leaf water potential to maintain osmotic homeostasis [24], as well as maintaining stomatal opening, thereby improving water uptake under water-scarce conditions. It is important to highlight that the observed reduction in EL (Figure 1B) may be related to lower lipid peroxidation of cellular membranes, as salicylic acid contributes to maintaining oxidative homeostasis, the balance between the production and elimination of reactive oxygen species (ROS) [25], thus preserving membrane integrity and preventing electrolyte leakage.

A greater stomatal opening (Figure 2B) was observed in plants subjected to foliar application of salicylic acid (SA), particularly when combined with deficit irrigation during the flowering stage (IS3). This effect may be associated with the role of SA in biochemical and physiological processes that favor the maintenance of stomatal aperture, possibly due to the reduction in ethylene and abscisic acid (ABA) levels, hormones known to promote stomatal closure under stress conditions [26]. With higher *gs* (Figure 2B), an increase in *E* (Figure 2C) was observed, resulting in a greater CO_2_ assimilation rate (Figure 2D). These results are likely associated with the strong positive correlation (*r* ≥ 0.85) among these variables, as evidenced in the Pearson correlation matrix for the 1.0 mM salicylic acid treatment (Figure 6B) and further supported by the principal component analysis (Figure 7).

The increase in stomatal conductance likely enhanced the diffusion of atmospheric carbon into the substomatal chamber, resulting in higher CO_2_ assimilation rates (Figure 2D) in sour passion fruit plants. This indicates that the fixed carbon was effectively used in the Calvin–Benson cycle, thereby reducing photorespiration and the excessive production of reactive oxygen species (ROS). Similarly, an increase in carboxylation efficiency (CEi) (Figure 2E) was observed in sour passion fruit plants, demonstrating that SA can improve the efficiency of the photosynthetic process. Moreover, a positive correlation between *gs* and *CEi* was observed in the Pearson correlation matrix for passion fruit plants treated with salicylic acid (Figure 6B). This finding supports the hypothesis that salicylic acid, as a signaling molecule, may play a crucial role in plant defense mechanisms through the activation of genes and pathways related to plant acclimation to water stress [27].

Nazar et al. [28], in a study on mustard plants under water stress, observed that foliar application of salicylic acid can positively regulate the synthesis of dehydrins, alter protein kinase activity, and modify RuBisCO and chlorophyll contents, thereby enhancing crop productivity under abiotic stress conditions. The results obtained in the present study reinforce the hypothesis that salicylic acid acts by inducing enzymes and proteins associated with the biosynthesis of secondary metabolites and cellular defense [29], thus promoting greater photosynthetic efficiency. These physiological and metabolic changes in plants contribute to the induction of drought stress tolerance.

In a study conducted by Lobato et al. [30], evaluating tomato plants under water deficit, a positive correlation was found between increased net photosynthesis and antioxidant defense following the application of 0.1 mM salicylic acid, particularly in the activities of superoxide dismutase, catalase, peroxidase, and ascorbate peroxidase. These enzymes play a crucial role in eliminating excess reactive oxygen species (ROS) and consequently reducing metabolic damage in plants.

Consistent with these findings, Fatima et al. [14], when assessing the concentrations and application methods of salicylic acid in yellow passion fruit seedlings under water deficit, reported that foliar application of salicylic acid at concentrations up to 1.3 mM increased stomatal conductance, transpiration, and CO_2_ assimilation rate, while reducing internal carbon concentration in the seedlings. This result may be associated with the effect of salicylic acid (SA) as a phytohormone, acting directly in the maintenance of the photosynthetic apparatus, possibly through enhanced photochemical activity and greater CO_2_ diffusion promoted by stomatal opening [31], thus preventing excessive oxygenase activity.

Deficit irrigation, regardless of the phenological stage, did not significantly affect the photosynthetic pigment content in yellow passion fruit plants. This result indicates that the water deficit imposed by the irrigation strategies did not compromise the functional integrity of photosystem II, thereby allowing the maintenance of light energy conversion into chemical energy [10].

However, with the application of SA, increases were observed in chlorophyll *a* (Figure 3A) and total chlorophyll (Figure 3B) contents, particularly under the IS2 and IS3 irrigation strategies, corresponding to 50% of the crop evapotranspiration (ETr). Under water deficit conditions, plants generally exhibit inhibition of chlorophyll synthesis and activation of the enzymatic antioxidant system as a defense mechanism against photo-oxidative damage [32]. Nevertheless, the application of SA prevented the degradation of photosynthetic pigments, contributing to the protection of the photosynthetic apparatus by reducing the accumulation of reactive oxygen species (ROS) and promoting osmotic adjustment [33], thereby enhancing water and nutrient absorption and protecting the photosynthetic machinery.

Salicylic acid (SA), when applied foliarly, can stimulate the activity of antioxidant enzymes that play a crucial role in the elimination of reactive oxygen species (ROS) and in the stabilization of thylakoid membranes. Consequently, SA-treated plants exhibited better preservation of chloroplast integrity, minimizing thylakoid depletion and lumen deformation, as also reported by [30,34], thus favoring their photochemical activity. These findings reinforce the role of SA as a modulator of chlorophyll stability and photosynthetic performance under deficit irrigation conditions.

Corroborating the results obtained for photosynthetic pigments, chlorophyll *a* fluorescence (Figure 4B–D) and stem diameter (Figure 5A) of yellow passion fruit under deficit irrigation also showed values similar to or higher than those observed in fully irrigated plants. These results indicate that the irrigation strategies applied during different phenological stages did not compromise photosynthetic efficiency and plant growth [35]. Possibly, because the photosynthetic apparatus remained intact when yellow passion fruit plants were subjected to water deficit during their phenological stages, total fruit production (Figure 5C) was not affected by the water restriction.

The reduction in initial fluorescence values (Figure 4A) in plants subjected to foliar application of salicylic acid, together with the increase in maximum fluorescence (Figure 4B) under irrigation with 50% of ETr during the flowering stage (IS3), possibly indicates greater efficiency in the conversion of light energy into chemical energy by photosystem II (PSII), thus enhancing water photolysis and resulting in higher photochemical efficiency. This result may be associated with reduced degradation of the D1 protein, an essential component of the PSII reaction center, and with the mitigation of oxidative damage, thereby supporting the functional integrity of the photosynthetic apparatus and optimizing the photosynthetic performance of the plants [36].

Roque et al. [37], when evaluating the foliar application of salicylic acid as a mitigator of water deficit in different guava genotypes, found that the application of 2.4 mM SA increased the content of photosynthetic pigments and chlorophyll *a* fluorescence in plants irrigated at 50% of ETr. This suggests that SA altered the chlorophyll fluorescence pattern by enhancing electron transport through the photosystems and increasing energy dissipation via light-independent photoprotective pathways [38].

The increase in stem diameter (Figure 5A) and fruit yield (Figure 5C) in sour passion fruit plants subjected to foliar application of salicylic acid (SA) may be associated with the higher relative water content in the leaf blade (Figure 1A), which resulted in greater cell turgor, thereby favoring cell elongation and expansion and consequently promoting plant growth. In addition, the enhancement in stomatal aperture (Figure 2B) directly influenced the CO_2_ assimilation rate (Figure 2D), since the diffusion of atmospheric carbon was intensified, promoting greater CO_2_ entry into the Calvin-Benson cycle and, consequently, increasing the instantaneous carboxylation efficiency (Figure 2E).

This set of physiological responses culminated in a higher production of photoassimilates, which were allocated to vegetative growth and fruit development (Figure 5C), especially in plants under the IS3 irrigation strategy combined with 1.0 mM foliar SA application. At appropriate concentrations, and depending on the phenological stage and method of application, salicylic acid can modulate plant physiological and biochemical processes, reducing oxidative damage caused by abiotic stresses and thereby promoting greater plant growth and productivity [39].

When evaluating the productivity of sweet cherry (*Prunus avium* L.) cv. Lapins subjected to deficit irrigation (60 and 100% of ETc) and foliar applications of salicylic acid (0 and 0.5 mM), González-Villagra et al. [40] found that irrigation at 60% of ETc reduced cherry yield; however, this effect was mitigated by the application of SA, resulting in approximately a 9% increase in productivity compared to plants not treated with the acid under the same irrigation conditions.

In general, unlike the other strategies, deficit irrigation during the flowering stage (IS3) showed greater acclimation to the imposed conditions, corroborating the findings of Fatima et al. [10] in sour passion fruit subjected to deficit irrigation at different phenological stages. According to the authors, the plants maintained photosynthetic activity, and the flowering stage promoted greater acclimation since the duration of water deficit was not sufficient to cause significant alterations in the metabolic pathways of sour passion fruit. These results are also reflected in the total fruit production (Figure 5C).

## 4. Materials and Methods

### 4.1. Location of the Experimental Site

**Figure 8:**
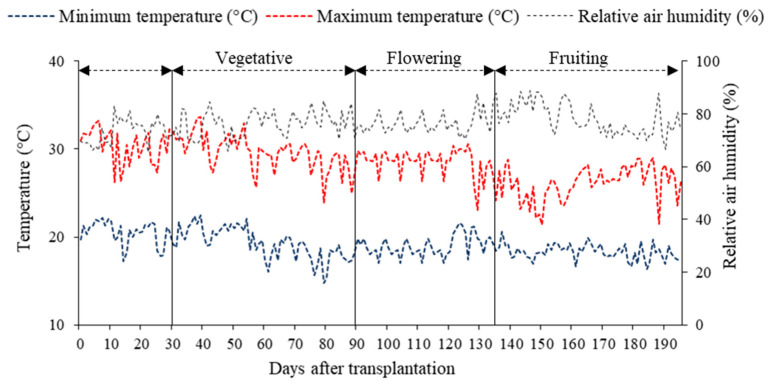
Maximum and minimum temperature and relative air humidity inside the greenhouse from 27 December 2023 to 4 August 2024.

The experiment was conducted from December 2023 to August 2024 in a protected environment belonging to the Academic Unit of Agricultural Engineering (UAEA) at the Federal University of Campina Grande (UFCG), located in the municipality of Campina Grande city, PB, Brazil, at coordinates 07°15′18″ S latitude, 35°52′28″ W longitude, and an average altitude of 550 m. The greenhouse was of the hoop type, measuring 30 m in length and 21 m in width, with a ceiling height of 3.0 m, and covered with low-density polyethylene (150 microns). Maximum and minimum temperatures, as well as relative air humidity during the experiment, are shown in Figure 8. The average temperature ranged from 14.80 to 33.66 °C, while the relative humidity fluctuated between 65.51 and 89.06%.

### 4.2. Experimental Design and Treatments

An experimental design in randomized blocks was adopted, arranged in a 6 × 2 factorial scheme, consisting of six deficit irrigation strategies based on the crop’s reference evapotranspiration (ETr)—full irrigation at 100% ETr throughout the crop cycle—IS1 (0–190 DAT); deficit irrigation at 50% ETr during the vegetative phase—IS2 (30–90 DAT); deficit irrigation at 50% ETr during the flowering phase—IS3 (90–135 DAT); deficit irrigation at 50% ETr during the fruiting phase—IS4 (135–190 DAT); deficit irrigation at 50% ETr during the successive vegetative/flowering phases—IS5 (30–135 DAT) and deficit irrigation at 50% ETr during the vegetative/fruiting phases—IS6 (30–190 DAT)—and without or with foliar application of salicylic acid (SA, 0 and 1 mM), with four repetitions, and one plant per plot, making a total of 48 experimental units. The salicylic acid concentrations were established based on the study by Sobrinho et al. [15], which evaluated foliar applications of SA in sour passion fruit irrigated with saline water (electrical conductivity ranging from 0.8 to 4.0 dS m^−1^).

### 4.3. Experimental Setup and Conduction

Seedlings were produced using three seeds of sour passion fruit (*Passiflora edulis Sims* f. flavicarpa Deg.) cv. Redondo Amarelo, sown in plastic bags measuring 15 × 20 cm, with a capacity of 3 kg, filled with a substrate composed of 84% soil, 15% sand, and 1% worm humus. After emergence, two plants per bag were thinned. Subsequently, staking was performed to maintain erect growth and prevent lodging.

At 80 days after sowing (DAS), the seedlings were transplanted into 200 L plastic pots equipped with drainage lysimeters using 20 mm diameter transparent drains. Each lysimeter was lined with a geotextile mat (Bidim type) and layered with 1 kg of crushed stone (No. 0) to prevent drain clogging. Below each lysimeter, two 2 L plastic bottles were attached to collect the drained water, allowing estimation of plant water consumption.

Subsequently, the pots were filled with 250 kg of Psamment soil collected from a depth of 0–30 cm in the municipality of Riachão do Bacamarte, PB, Brazil. The soil’s physicochemical and water characteristics were determined following the methodology described by Teixeira et al. [41] and were as follows: pH 5.40; organic matter = 17.42 g dm^−3^; phosphorus (P) = 2.92 mg dm^−3^. Exchangeable cation contents were potassium (K) = 0.28 cmolc kg^−1^, sodium (Na) = 0.04 cmolc kg^−1^, calcium (Ca^2+^) = 1.87 cmolc kg^−1^, and magnesium (Mg^2+^) = 1.70 cmolc kg^−1^. Acidity-related elements were aluminum (Al^3+^) = 0.20 cmolc kg^−1^ and hydrogen plus aluminum (H^+^ + Al^3+^) = 2.88 cmolc kg^−1^. Regarding physical properties, particle size analysis showed 675.2 g kg^−1^ sand, 221.8 g kg^−1^ silt, and 103 g kg^−1^ clay. The soil bulk density was 1.51 g cm^−3^, and soil moisture was 5.34 dag kg^−1^.

Irrigation was carried out daily at 7:00 a.m., according to the established treatments. The volume of water applied to each container was determined based on the water balance. The deficit irrigation equivalent to 50% of the reference evapotranspiration (ETr) was applied according to the crop development stage. To assess soil water content, soil samples were collected at each transition between phenological stages from depths of 0–20 and 20–40 cm, with the aim of determining water content using the gravimetric method [42]. The definition of these stages was based on morphological criteria: the vegetative state (30–90 DAT) spanned from transplanting to the emergence of the floral primordium; the flowering stage (90–136 DAT) covered the period from the appearance of the floral primordium to anthesis; and the fruiting stage (136–196 DAT) extended from the beginning of fruit formation to the onset of partial yellowing of the fruits. Stage transitions were determined when 50% of the plants exhibited phenological characteristics corresponding to the subsequent stage, as described by Pinheiro et al. [43].

The irrigation management strategies (Table 6) with water deficit began at 30 days after transplanting (DAT), when the plants reached a height of 130 cm.

The salicylic acid (SA) solution was prepared for each application event by diluting it in 30% ethyl alcohol, due to its low solubility in water at room temperature. To reduce the surface tension of the droplets on the leaf surface, the adjuvant Wil Fix was added at a concentration of 0.5 mL L^−1^ of solution. Applications of SA began 20 days after transplanting (DAT) by spraying both the abaxial and adaxial leaf surfaces at 15-day intervals, between 5:00 p.m. and 6:00 p.m., using a Jacto backpack sprayer, model Jacto XP, with a 12 L capacity, maximum working pressure of 88 psi (6 bar), and a JD 12P nozzle. In total, each plant received 5 applications up to the flowering stage, totaling 2.2 L of solution per plant. To prevent drift of the salicylic acid during application, a plastic curtain was used to cover the entire plant, avoiding unintended application to other treatments.

Fertilization was carried out according to the recommendations of Costa et al. [44] specifically for passion fruit cultivation. The sources used were urea (45% N) as the nitrogen source; single superphosphate (18% P_2_O_5_, 16% Ca^2+^, and 10% S) as the phosphorus source; and potassium chloride (60% K_2_O) as the potassium source. Phosphorus was applied as a single dose of 120 g per plant. Nitrogen and potassium fertilization began 15 days after transplanting (DAT) and was applied every 15 days through fertigation. During the vegetative and flowering phases, 166.57 g of N and 156.58 g of K_2_O were applied per plant. In the fruiting phase, the doses applied were 140.00 g of N and 360.00 g of K_2_O. Micronutrients were supplied via foliar application every 15 days throughout the cycle, using a backpack sprayer with a solution containing 1.0 g L^−1^ of 1.2% Mg, 0.85% B, 3.4% Fe, 4.2% Zn, 3.2% Mn, 0.5% Cu, and 0.06% Mo.

**Figure 9:**
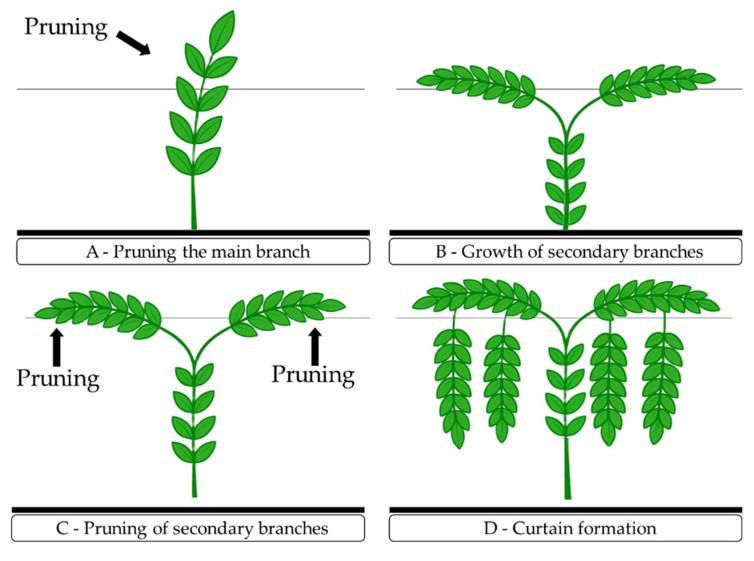
Training pruning of sour passion fruit: cutting of the primary branch (**A**); guiding of secondary branches along the trellis system (**B**); cutting of secondary branches (**C**); and pruning of tertiary branches (**D**).

The vertical trellis training system was implemented in the experimental setup using the greenhouse structure and galvanized wire (No. 14). The support wire was positioned at a height of 2.00 m from the greenhouse floor, resulting in a 2.0 m tall curtain. Plants were trained with a single stem, tied with 10 mm wide polypropylene twine, to grow upright until reaching the trellis height. When the plants exceeded the trellis by 10 cm, the apical bud was pruned to promote the growth of two secondary branches, which were directed one to each side until reaching 1.0 m in length. After the secondary branches reached this length, a new apical bud pruning was performed to stimulate the emergence of tertiary branches, which were trained to reach 30 cm above the soil. During the experiment, tendrils and sucker shoots were removed to favor the full development of the crop, following the procedures described by Pinheiro et al. [43], as can be seen in Figure 9.

Pollination was carried out manually until flower production ceased. During the experiment, weeding, soil scarification, and phytosanitary controls recommended for the crop were performed, as well as monitoring for the appearance of pests and diseases, with control measures implemented when necessary. Chemical pesticides with the active ingredients Abamectin and Chlorfenapyr were used to control aphids (*Aphis gossypii*), scale insects (*Phenacoccus solenopsis*), whitefly (*Bemisia tabaci*), passion fruit butterfly (*Dione juno juno*), and green stink bug (*Nezara viridula*), applied via spraying whenever needed.

### 4.4. Traits Analyzed

At 160 days after transplanting (DAT), the following variables were evaluated: relative water content—RWC (%), electrolyte leakage—EL (%) in the leaf blade, CO_2_ assimilation rate—*A* (µmol CO_2_ m^−2^ s^−1^), stomatal conductance—*gs* (mol H_2_O m^−2^ s^−1^), transpiration—*E* (mol H_2_O m^−2^ s^−1^), internal CO_2_ concentration—*Ci* (µmol CO_2_ m^−2^ s^−1^), instantaneous carboxylation efficiency—*A*/*Ci* (*CEi*) [(µmol CO_2_ m^−2^ s^−1^)/(mol CO_2_ m^−2^ s^−1^)], instantaneous water use efficiency—*WUEi* (A/E) [(µmol CO_2_ m^−2^ s^−1^)/(mol H_2_O m^−2^ s^−1^)], chlorophyll *a* content—Chl *a* (μg mL^−1^), chlorophyll *b*—Chl *b* (μg mL^−1^), total chlorophyll—Chl t (μg mL^−1^), carotenoids—Car (μg mL^−1^), initial fluorescence—Fo, maximum fluorescence—Fm, variable fluorescence—Fv, and quantum efficiency of photosystem II—Fv/Fm, stem diameter—SD (cm), and mean secondary branch diameter—SDRM (cm). Fruit yield per plant—PT (g per plant) was assessed from 156 to 196 DAT.

The relative water content (RWC%) was determined following the methodology of Weatherley [45]. Five 12 mm leaf discs were collected from the middle third of the main branch to obtain the fresh mass. The discs were then immersed in 50 mL of distilled water in beakers for 24 h at room temperature. After this period, excess water was removed using paper towels to obtain the turgid mass of the samples. Subsequently, the discs were dried at approximately 65 ± 3 °C until reaching a constant mass to determine the dry weight.

Electrolyte leakage (EL%) was determined using a copper punch to obtain, for each experimental unit, five leaf discs of known area. The discs were washed and placed in Erlenmeyer flasks containing 50 mL of distilled water. After sealing with aluminum foil, the flasks were kept at 25 °C for 24 h, after which the initial electrical conductivity of the medium was measured using a bench conductivity meter. Subsequently, the flasks were heated at 90 °C for 120 min in a drying oven, and the final electrical conductivity was measured. Electrolyte leakage was expressed as the percentage of conductivity relative to the total conductivity after the 120 min treatment at 90 °C, following the methodology proposed by Scotti-Campos et al. [46].

The gas exchange variables were evaluated using the portable photosynthesis measurement system “LCPro+” from ADC Bio Scientific Ltd. (Hoddesdon, UK), with temperature controlled at 25 °C, irradiance of 1200 µmol photons m^−2^ s^−1^, airflow of 200 mL min^−1^, and ambient CO_2_ level, determined through a photosynthetic light saturation curve assessed on the third leaf from the apex, between 7:00 and 9:30 a.m. In determining gas exchange, an environment with an average irradiance of 882.00 µmol photons m^−2^ s^−1^ and a photoperiod of approximately 11 h for the passion fruit plant was considered [44].

The contents of chlorophyll *a*, chlorophyll *b*, total chlorophyll, and carotenoids were determined based on the methodology adapted from Arnon [47], using plant extracts obtained from leaf disc samples with an area of 12 mm, collected from the third fully expanded leaf from the apical bud. Each sample received 5 mL of dimethyl sulfoxide and was stored at room temperature. After 48 h, a spectrophotometer was used to read the photosynthetic pigments at absorbance wavelengths of 470, 647, and 663 nm. The values obtained for chlorophyll *a*, *b*, total chlorophyll, and carotenoids in the leaves were expressed in μg mL^−1^.

Chlorophyll *a* fluorescence was measured on the same leaf used for gas exchange assessments, using a modulated pulse fluorometer (OS5p, Opti-Science, Hudson, NY, USA) following the Fv/Fm protocol. This allowed determination of the fluorescence induction variables: initial fluorescence (F_0_), maximum fluorescence (Fm), variable fluorescence (Fv = Fm − F_0_), and the quantum efficiency of photosystem II (Fv/Fm). The protocol was conducted after dark adaptation of the leaves for 30 min, using the fluorometer clip to ensure that all electron acceptors were oxidized, i.e., with reaction centers fully open.

The stem diameter (SD) was measured directly using a caliper 3 cm above the plant collar. The average diameter of the secondary branches (SDRM) was measured at 3 cm from the base of each branch. The production component of the sour passion fruit was assessed by directly weighing the fruits after fully maturing, which exhibited a completely yellow peel, from 136 to 196 days after sowing (DAS).

### 4.5. Statistical Analysis

The collected data were initially subjected to normality (Shapiro–Wilk) and homoscedasticity (Levene) tests. For the irrigation strategies with water deficit, the Scott-Knott test (*p* ≤ 0.05) was applied, while for the salicylic acid (SA) concentrations, the Tukey test (*p* ≤ 0.05) was used. Subsequently, if the data presented a normal distribution, Pearson’s correlation analysis (*p* ≤ 0.05) was performed for each concentration (0.0 and 1.0 mM). A principal component analysis was also performed, retaining only variables with a correlation coefficient above 0.60 [25]. All statistical analyses were conducted using RStudio software (version 4.1.0), with the support of the AgroR, corrplot, ggcorrplot, and mclust packages.

## 5. Conclusions

Water deficit conditions affected the plants to varying degrees, particularly when passion fruit plants were irrigated with 50% of the ETr during the vegetative, fruiting, vegetative/flowering, and vegetative/fruiting stages. The latter was the most detrimental among the irrigation strategies, as it increased the percentage of electrolyte leakage while reducing gas exchange and the content of photosynthetic pigments. However, water deficit conditions did not affect the growth and yield of passion fruit.

The flowering stage exhibited greater morphophysiological acclimation under deficit irrigation conditions. Nevertheless, the foliar application of salicylic acid at 1.0 mM enhanced gas exchange, photosynthetic efficiency, relative water content, and productivity. In addition, it contributed to the reduction in electrolyte leakage in leaf tissues. These results indicate that the application of salicylic acid at a concentration of 1.0 mM is a promising strategy to mitigate the effects of water deficit in passion fruit, supporting the maintenance of physiological activities and productive performance under limited water availability.

Salicylic acid is an easily accessible and low-cost compound, soluble in hot water, with the potential to induce physiological mechanisms of tolerance to abiotic stresses. However, its beneficial effects on passion fruit cultivation still present several gaps in knowledge. Therefore, further research is required to elucidate the biochemical and nutritional mechanisms through which salicylic acid (SA) may induce tolerance to water deficit.

Moreover, field validation is crucial to consolidate the findings and provide more comprehensive insights into the behavior of passion fruit in response to salicylic acid application as a strategy to mitigate water deficit stress across different phenological stages.

## Figures and Tables

**Table 1:** Summary of the analysis of variance for relative water content (RWC%) and electrolyte leakage (EL%) in the leaf blade of sour passion fruit cv. Redondo Amarelo, as affected by the interaction between irrigation strategies under water deficit and salicylic acid application, at 160 days after transplanting.

Source of Variation	DF	Mean Squares	
		RWC%	EL%
Irrigation strategies (IS)	5	51.98 **	1.57 × 10^6^ **
Salicylic acid (SA)	1	0.80 **	5.96 × 10 *
Interaction (IS × SA)	5	28.98 **	4.70 × 10^4^ ***
Blocks	3	8.34 ^ns^	6.70 × 10^4 ns^
Residue	33	7.84	9.00 × 10^4^
CV (%)		3.81	9.41

DF—Degrees of freedom; CV—Coefficient of variation; *, **, ***, ^ns^—Significant at *p* ≤ 0.05, *p* ≤ 0.01, *p* ≤ 0.001, and not significant, respectively.

**Table 2:** Summary of the analysis of variance for intercellular CO_2_ concentration (*Ci*), stomatal conductance (*gs*), transpiration rate (*E*), CO_2_ assimilation rate (*A*), instantaneous carboxylation efficiency (*CEi*), and instantaneous water use efficiency (*WUEi*) of sour passion fruit cv. Redondo Ama-relo cultivated under different irrigation strategies with water deficit and salicylic acid application, at 160 days after transplanting (DAT).

Source of Variation	DF	Mean Squares					
		*Ci*	*gs*	*E*	*A*	*CEi*	*WUEi*
Irrigation strategies (IS)	5	2408.14 ^ns^	0.0041 ***	1.97 ***	29.27 ***	6 × 10^−4^ ***	2.29 ***
Salicylic acid (SA)	1	5104.68 **	0.0012 *	0.54 ***	16.93 ***	8 × 10^−4^ ***	0.32 ^ns^
Interação (IS × SA)	5	1042.18 ^ns^	0.0002 ***	0.037 **	6.78 ***	4 × 10^−4^ ***	1.09 **
Blocks	3	167.39 ^ns^	0.0001 ^ns^	0.005 ^ns^	0.16 ^ns^	1.7 × 10^−4^ **	0.38 ^ns^
Residue	33	7563.85	0.00024	0.012	0.26	7.99 × 10^−4^	0.08
CV (%)		6.39	12.66	5.64	5.95	7.67	6.77

DF—Degrees of freedom; CV—Coefficient of variation; *, **, ***, ^ns^—Significant at *p* ≤ 0.05, *p* ≤ 0.01, *p* ≤ 0.001, and not significant, respectively.

**Table 3:** Summary of the analysis of variance for chlorophyll *a* (Chl *a*), chlorophyll *b* (Chl *b*), total chlorophyll (Chl t), and carotenoids (Car) of sour passion fruit cv. Redondo Amarelo cultivated under deficit irrigation strategies and salicylic acid application, at 160 days after transplanting.

Source of Variation	DF	Mean Squares			
		Chl *a*	Chl *b*	Chl t	Car
Irrigation strategies (IS)	5	19.57 × 10^6^ ***	1.57 × 10^6 ns^	3.57 × 10^7^ **	1.28 × 10^4^ **
Salicylic acid (SA)	1	4.84 × 10^4^ **	5.96 × 10^4 ns^	2.45 × 10^7^ ***	4.43 × 10^5^ **
Intertaction (IS × SA)	5	19.33 × 10^5^ **	4.70 × 10^4 ns^	1.64 × 10^5^ **	1.02 × 10^4^ **
Blocks	3	4.50 × 10^3 ns^	6.70 × 10^4 ns^	1.64 × 10^4 ns^	2.47 × 10^3^ **
Residue	33	7.38 × 10^3^	9.00 × 10^4^	2.53 × 10^4^	2.57 × 10^5^ **
CV (%)		3.16	8.63	6.36	12.7

DF—Degrees of freedom; CV—Coefficient of variation; **, ***, ^ns^—Significant at *p* ≤ 0.01, *p* ≤ 0.001, and not significant, respectively.

**Table 4:** Summary of the analysis of variance for initial fluorescence (F_o_), maximum fluorescence (Fm), variable fluorescence (Fv), and quantum efficiency of photosystem II (Fv/Fm) of sour passion fruit cv. Redondo Amarelo cultivated under deficit irrigation strategies and salicylic acid application, at 160 days after transplanting.

Source of Variation	DF	Mean Squares			
		F_o_	Fm	Fv	Fv/Fm
Irrigation strategies (IS)	5	273.88 ^ns^	2524.73 ^ns^	1845.85 *	0.0023 *
Salicylic acid (SA)	1	2286.33 **	1496.35 ^ns^	1170.18 ^ns^	0.0011 ^ns^
Interaction (IS × SA)	5	348.18 ^ns^	13614.20 *	7332.54 ^ns^	0.0016 ^ns^
Blocks	3	288.39 ^ns^	7144.16 ^ns^	4205.18 ^ns^	0.0016 ^ns^
Residue	33	298.85	5335.86	3584.56	0.0008
CV (%)		5.83	6.05	6.27	3.7

DF—Degrees of freedom; CV—Coefficient of variation; *, **, ^ns^—Significant at *p* ≤ 0.05, *p* ≤ 0.01, and not significant, respectively.

**Table 5:** Summary of the analysis of variance for the diameter of the stem of the main branch (SD), mean diameter of the secondary branches (SDSR) at 160 days after transplanting, and the production per plant (TP) of the sour passion fruit cv. Redondo Amarelo from 136 to 196 days, cultivated under irrigation strategies with water deficit and the application of salicylic acid.

Source of Variation	DF	Mean Squares		
		SD	SDSR	TP
Irrigation strategies (IS)	5	0.74 ^ns^	0.50 ^ns^	8.60 **
Salicylic acid (SA)	1	14.49 ***	3.48 *	43.23 *
Interação (IS × SA)	5	3.92 *	0.48 ^ns^	5.89 **
Blocks	3	0.52 ^ns^	0.32 ^ns^	2.9 ^ns^
Residue	33	0.81	0.62	1.23
CV (%)		5.0	8.06	5.32

DF—Degrees of freedom; CV—Coefficient of variation; *, **, ***, ^ns^—Significant at *p* ≤ 0.05, *p* ≤ 0.01, *p* ≤ 0.001, and not significant, respectively.

**Table 6:** Water consumption of *Passiflora edulis* f. *flavicarpa* cv. Redondo Amarelo under different irrigation strategies, estimated by drainage lysimetry.

Irrigation Strategy	Water Consumption (mm)	
	A1	A2
Full irrigation—IS1	1483.55	1384.03
Vegetative phase—IS2	1251.04	1285.45
Flowering phase—IS3	1284.01	1185.86
Fruiting phase—IS4	1046.26	1119.16
Vegetative + Flowering phases—IS5	1063.57	1097.71
Vegetative + Fruiting phases—IS6	918.28	892.36

IS1—Irrigation with 100% of the reference evapotranspiration (ETr) throughout the entire crop cycle; IS2—Irrigation with 50% ETr during the vegetative phase; IS3—Irrigation with 50% ETr during the flowering phase; IS4—Irrigation with 50% ETr during the fruiting phase; IS5—Successive irrigation deficits during the vegetative/flowering phases; IS6—Successive irrigation deficits during the vegetative/fruiting phases. A1—Plants that did not receive foliar application of salicylic acid; A2—Plants that received foliar application of 1.0 mM of salicylic acid. Area of the pot used: 0.180 m^2^.

## Data Availability

The original contributions presented in this study are included in the article. Further inquiries can be directed to the corresponding author.
